# A Rapid Transcriptome Response Is Associated with Desiccation Resistance in Aerially-Exposed Killifish Embryos

**DOI:** 10.1371/journal.pone.0064410

**Published:** 2013-05-31

**Authors:** Angèle Tingaud-Sequeira, Juan-José Lozano, Cinta Zapater, David Otero, Michael Kube, Richard Reinhardt, Joan Cerdà

**Affiliations:** 1 Institut de Recerca i Tecnologia Agroalimentàries (IRTA)-Institut de Ciències del Mar, CSIC, Barcelona, Spain; 2 Bioinformatics Platform, CIBERHED, Barcelona, Spain; 3 Max Planck Institute for Molecular Genetics, Berlin-Dahlem, Germany; Auburn University, United States of America

## Abstract

Delayed hatching is a form of dormancy evolved in some amphibian and fish embryos to cope with environmental conditions transiently hostile to the survival of hatchlings or larvae. While diapause and cryptobiosis have been extensively studied in several animals, very little is known concerning the molecular mechanisms involved in the sensing and response of fish embryos to environmental cues. Embryos of the euryhaline killifish *Fundulus heteroclitus* advance dvelopment when exposed to air but hatching is suspended until flooding with seawater. Here, we investigated how transcriptome regulation underpins this adaptive response by examining changes in gene expression profiles of aerially incubated killifish embryos at ∼100% relative humidity, compared to embryos continuously flooded in water. The results confirm that mid-gastrula embryos are able to stimulate development in response to aerial incubation, which is accompanied by the differential expression of at least 806 distinct genes during a 24 h period. Most of these genes (∼70%) appear to be differentially expressed within 3 h of aerial exposure, suggesting a broad and rapid transcriptomic response. This response seems to include an early sensing phase, which overlaps with a tissue remodeling and activation of embryonic development phase involving many regulatory and metabolic pathways. Interestingly, we found fast (0.5–1 h) transcriptional differences in representatives of classical “stress” proteins, such as some molecular chaperones, members of signalling pathways typically involved in the transduction of sensor signals to stress response genes, and oxidative stress-related proteins, similar to that described in other animals undergoing dormancy, diapause or desiccation. To our knowledge, these data represent the first transcriptional profiling of molecular processes associated with desiccation resistance during delayed hatching in non-mammalian vertebrates. The exceptional transcriptomic plasticity observed in killifish embryos provides an important insight as to how the embryos are able to rapidly adapt to non-lethal desiccation conditions.

## Introduction

Arrested development is a form of dormancy in which metabolic activity is significantly depressed or even absent. It is a widespread strategy employed by many organisms, from prokaryotes to mammals, in response to unfavorable thermal, nutritional or hydration conditions [1]. Dormancy encompasses the phenomena of diapause, quiescence or cryptobiosis [2], [3], [4], [5], and can be associated with desiccation (i.e. anhydrobiosis) when long-term periods of metabolic arrest are needed for survival [6]. Interestingly, however, recent studies suggest that the molecular pathways underlying the process of dormancy show important similarities among different organisms, in spite of their very different survival strategies [1], [5].

In fish, embryonic dormancy is the most widespread form of arrested development and is often associated with dehydration tolerance, which allows survival during transient or prolonged environmental hypoxia and anoxia [7], [8]. Three major forms of arrested development have been described for fish embryos: delayed hatching, embryonic diapause, and anoxia-induced quiescence [7]. Diapause is very common among annual killifishes (Cyprinidontiformes) which inhabit ephemeral ponds in regions of Africa and South and Central America that experience annual dry and rainy seasons [7], [9]. In annual killifish, diapause may occur at three distinct developmental stages, diapause I, II and III [10], which appear to respond to different environmental cues for induction and breakage of dormancy (reviewed by [8]). Studies on diapause II (occurring after neurulation and somitogenesis, but prior to initiation of the major phases of organogenesis) and anoxia-induced quiescence embryos of the annual killifish *Austrofundulus limnaeus* show that during diapause metabolism is supported using anaerobic metabolic pathways, regardless of oxygen availability, and high ATP and a positive cellular energy status, whereas anoxia causes a severe reduction in ATP content and large reductions in adenylate energy charge [8]. In addition, in response to hypoxia-induced diapause, most cells become arrested in the G1/G0 phase of the cell cycle which may favour genome integrity for the recovery phase [8].

Delayed hatching is observed in both fish and amphibians and is typically associated with the deposition of eggs in an aerial environment [7], [11], [12], [13], [14], [15]. In contrast to diapause, delayed hatching seems to result in a reduced, but not arrested rate of metabolism and development [7]. Comparison of hatching across teleostean taxa indicates great variability in the stage at hatching and in the duration of incubation [13], and therefore the plasticity for hatching time is likely linked to the embryo’s ability to sense environmental cues [14]. An extensively studied fish model of delayed hatching is the common mummichog, *Fundulus heteroclitus*, a marine, non-migratory killifish typically inhabiting coastal marshes and inland systems [16], [17], [18], [19], [20], [21]. During the reproductive cycle of this species, gonad maturity and spawning readiness coincide with new and full moons, and spawning is thereby synchronized with the semi-lunar cycle of tides in the tide marsh habitat [22], [23]. Eggs are laid in multiple clutches at the high water mark during the high spring tides associated with new and full moons, and embryos develop in air and hatch (generally in about 14 days) when the next spring tide floods them [18], [22], [23], [24]. Northern populations of *F. heteroclitus macrolepidus* of North America may spawn throughout the tidal cycle on each high tide [25], and thus also in this case embryos will possibly be exposed to aerial incubations conditions for at least 14 days [26]. It is thought that hypoxia caused by flooding with seawater is the major cue that initiates hatching [18], but the molecular mechanisms involved are not known.

Incubation of *F. heteroclitus* embryos in aerial conditions most likely expose the embryos to higher levels of oxygen and higher temperature, which result in enhanced developmental rates, advanced or higher hatching, and larger hatchlings, with respect to embryos constantly submerged in water [19], [20], [27], [28]. Therefore, delayed hatching in *F. heteroclitus* is not associated with the depression of metabolism. However, aerially incubated embryos are likely to be also exposed to desiccation and thermal stress, and possibly osmotic stress due to water loss [26]. Laboratory-controlled experiments suggest that the low permeability of membranes of the embryonic compartments prevents significant water loss and allows prolonged survival of embryos in dehydrated conditions, regardless whether the desiccation conditions are stressful or not [26], [27]. In aerially incubated embryos at ∼100% relative humidity (RH), Tingaud-Sequeira et al. [27] found that expression of aquaporin-3a (Aqp3a) is down-regulated and removed from the basolateral membrane of the enveloping layer (EVL) epithelium [29], which may account in part for the low permeability of the embryonic membranes during air exposure. These findings thus suggest that killifish embryos are able to transduce even moderate dehydration conditions into molecular responses within few hours of air exposure. However, in more severe desiccation conditions (∼23% RH) it has been hypothesized the role of additional mechanisms involving chaperone proteins such as heat shock proteins and compatible solutes such as free amino acids, which may help to stabilize vital cellular proteins [26].

Although the plasticity of hatching is well described for fish and amphibians, the molecular mechanisms involved in the sensing and response of embryos to environmental cues are largely unknown. It is recognized, however, that adult populations of *F. heteroclitus* exhibit both physiological and adaptive responses to cope with the variable environments they inhabit, in which variations in gene expression have been shown to play a role in the evolutionary adaptation to diverse environments [21], [30], [31], [32], [33], [34], [35], [36], [37], [38], [39]. Based on these and our previous studies, we hypothesized that killifish embryos may be able to rapidly transduce environmental desiccation conditions into systemic molecular responses, even when the conditions do not result in the dehydration of embryos. In a recent comprehensive study, Bozinovic et al. [40] have described the pattern of gene expression of nearly 7,000 genes during all 40 stages of embryogenesis in a North American population of *F. heteroclitus* providing statistical support for the temporal dynamics of developmental gene expression. However, information on transcriptomic changes in embryos in response to aerial incubation is still lacking. To test our hypothesis, in the present study we generated an expressed sequence tag (EST) database from water and aerially incubated killifish embryos by Sanger sequencing containing ∼11,000 unigenes, designed a microarray, and investigated gene expression profiles of mid-gastrula embryos incubated in air at ∼100% RH for 24 h in comparison with embryos continuously immersed in water.

## Materials and Methods

### Ethics Statement

Adult killifish, *F. heteroclitus*, were collected from saltmarshes in the bay of Cádiz (southern Spain) by local fishermen using cast nets and transported to the laboratory. Since *F. heteroclitus* is not considered an endangered species, no specific permissions were required to collect the specimens in Spain. All the procedures related to the care and use of the fish were approved by the Ethics Committee of the Institut de Recerca i Tecnologia Agroalimentàries (IRTA, Spain) in accordance with the “Guiding Principles for the Care and Use of Laboratory Animals”.

### Fish Maintenance and Egg Collection

Fish were maintained in the laboratory in 500-liter tanks as previously described [41]. The tanks were provided with aerated filtered natural seawater (38‰ salinity; 1,110 mOsmol/kg H_2_O) and maintained at 24–25°C under a daily photoperiod of 14∶10 (L:D). Fish were fed daily with commercial pellets (Alpha 3, INVE) and a supplement of fresh squid twice or three times a week. Fish started to spawn naturally after 1–2 weeks of acclimation, and benthic eggs were collected daily by means of plastic trays covered by a 3-mm net placed at the bottom of the tanks [27].

### Embryo Culture

Embryos were staged according to Armstrong and Child [42] and routinely incubated in 60-mm Petri dishes with filtered seawater at 24–25°C in a temperature-controlled incubator in the dark. The Spanish population of *F. heteroclitus* employed in the present work seem to exhibit significantly higher developmental rates than those of the southern genotypes of the North American populations employed by Bozinovic et al. [40]. Thus, blastula (stage 10–11), mid-gastrula (50% epiboly; stage 17), and late neurula (stage 20–21) stages were reached at approximately 3, 6 and 24 h post fertilization, respectively, in our population [27], whereas at the same temperature (24–25°C) the southern population of *F. heteroclitus* of North America reaches these embryonic stages at approximately 11, 31 and 42–44 h post fertilization, respectively [40].

For sample collection, groups of chorion-enclosed embryos (*n* = 25–30) at the 2–4 cells stage were incubated completely submerged in water or exposed to air for up to 14 days at 24–25°C as above. The dishes were placed in sealed plastic boxes containing wet cellulose paper to maintain approximately 100% RH. At 2–4 cells, morula, blastula, mid-gastrula, and neurula stages, as well as after 48 h, or 5, 7, and 14 days of incubation, samples of embryos were collected. In other trials, embryos incubated in air for 12 days from the blastula stage were transferred to seawater, and samples were collected after 10, 20 and 30 min, and 1 h when all embryos hatched [27]. In all cases, embryo samples were collected from different batches throughout the year. Samples were deep-frozen in liquid nitrogen, and stored at −80°C until RNA extraction.

### Construction of a Multi-stage *F. heteroclitus* cDNA Library and Functional Characterization of Sequence Data

Three groups of killifish embryos were prepared for cDNA library construction: (1) pool of embryos at different developmental stages maintained submerged in water; (2) pool of embryos at different developmental stages under aerial exposure; and (3) pool of air-exposed embryos collected at different times after water addition. Total RNA was extracted from each of these groups using the RNAeasy Mini Kit (Qiagen, Hilden, Germany) following the manufacturer’s instructions. An equal aliquot of total RNA from the three pools was mixed together and cDNA was synthesized and normalized using a combination of the SMART cDNA Library Construction kit (BD Biosciences Clontech, Palo-Alto, CA) and Trimmer-Direct kit (Evrogen, Moscow, Russia) according to the protocol from Evrogen (http://www.evrogen.com/trimmer-direct.pdf). Modifications to the protocol were made concerning the columns used for size selection and the cloning vector: to improve clone size selection Chroma spin 1000 was used instead of Chroma spin 400 (both BD Biosciences Clontech, Palo-Alto, CA), and pal32 (Evrogen, Moscow, Russia) was used for directional cloning with insertion between two *Sfi*I sites (GGCCATTACGGCCGGG del(CATGTC) GGCCGCCTCGGCC. This procedure was chosen because of the low amount of starting material [43], and the normalisation process increased the efficiency of rare transcript discovery [44], [45]. Plasmids were transferred via electroporation to *Escherichia coli* (strain DH10B, Invitrogen, Karlsruhe, Germany).

Plasmids were isolated from the master library according to the method of Hecht et al. [46], and were 5′ end Sanger-sequenced using Dye Terminator Chemistry version 3.1 (ABI, Weiterstadt, Germany) and 3730XL ABI capillary sequencer systems (ABI, Weiterstadt, Germany). Sequence fasta files were processed using the script Trace2dbest software [47], which incorporated the phred [48], [49] and crossmatch (P. Green, unpublished) softwares. A minimum cut-off value of 150 bp was applied after quality control processing for sequence database searching and for generating the submission file for the GenBank EST database (dbEST) [50]. The sequences are under accession numbers GT091489-GT107174. The TGI clustering tools (TGICL) software [51] was used for clustering the fasta files, incorporating quality scores.

Killifish singleton sequences and contigs were submitted for gene ontology (GO) [52] annotation to the online version of the BLAST2GO v1 program [53]. Annotated accession numbers and GO numbers were derived with NCBI’s QBLAST (http://blast.ncbi.nlm.nih.gov/Blast.cgi), with an expectation E-value ≤10^−3^ and an HSP length cut-off of 33. Contig sequences were then annotated according to the following parameters: a pre-E-value-Hit-Filter of 10^−6^, a pro-Similarity-Hit-Filter of 15, an annotation cut-off of 55, and a GO weight of 5. Directed acyclic graphs (DAG’s) were generated using a sequence filter of 5, an alpha score of 0.6 and a 0 node score filter. A score was computed at each node by Blast2GO to highlight areas of high annotation. The data presented represent the level 2 analysis, illustrating general functional categories.

### Construction of a Killifish cDNA Microarray

The complete killifish EST bank (16,896 clones) was amplified from bacterial templates via standard PCR as previously described [54]. Following amplification, 25 µl of PCR products was concentrated by isopropanol precipitation. The pellets were dried at room temperature, resuspended overnight in 15 µl of spotting buffer (3×SSC [1×SSC is 0.15 M NaCl, 10 mM sodium citrate], 1.5 M betaine [55]) on a rotary shaker and subsequently transferred to 384-well polystyrene spotting plates (Genetix). Size and concentration of the PCR products were verified by agarose gel electrophoresis and spectrophotometric measurements.

Spotting was performed with a modified Genetix Qarray spotting robot by using TeleChem (Sunnyvale) CMP4 split pins and TeleChem SuperAmine slides. After incubation for 12 h at 20°C and 55% relative humidity, the slides were snap dried on a heating plate (200°C) for 30 s. DNA was linked to the slide surface by UV irradiation with a Stratagene UV-Stratalinker (twice at 1,200 µJ). The slide surface was subsequently blocked as described previously [55]. Slides were stored at room temperature and protected from light and humidity until further use.

### Microarray Experimental Design and Hybridization

Groups of embryos (*n* = 25–30) at the mid-gastrula stage were incubated in the dark in seawater or in air at approximately 100% RH as indicated above. After 30 min, and 1, 3, 6, 12 and 24 h, embryos from each group were frozen and stored at −80°C. Samples of embryos at 2–4 cells and mid-gastrula stages from the same batches were also collected. Embryo samples used for microarray analysis were from three independent incubation experiments employing batches of embryos collected at different times during the year. Thus, three biological replicates were employed for microarray analysis. The RNA was extracted as indicated above, except that for 1–2 cells stage embryos the RNeasy Lipid Tissue Mini Kit (Qiagen) was used.

Total RNA (0.5 µg) from each group of embryos was amplified and labelled with fluorescent cyanine dye 3 (Cy3) employing the Ambion MessageAmp™ aRNA amplification kit (Applied Biosystems). RNA quality control and quantification and labeling efficiency was measured by agarose gel electrophoresis and fluorescence scanning of the gel with a Fuji FLA-8000 scanner at 532 and 640 nm followed by subsequent staining of the gel with ethidium bromide. Labelled cDNAs were purified by using Microcon YM-30 spin columns (Millipore). The Cy3-labelled cDNAs from three biological replicates were hybridized independently to the microarray. For each hybridization, the Cy3-labeled reference (gastrula embryos at 25–75% epiboly) and experimental sample were pooled and mixed with 2 µl of a DNA mixture consisting of 2.5 µg of herring sperm DNA (Invitrogen)/µl, 25 ng of acetylated bovine serum albumin (Promega)/µl, and 0.6 µM unlabeled oligonucleotides. The mixture was concentrated with a SpeedVac (volume of 3 to 5 µl), resuspended in 50 µl of DIG Easy Hyb buffer (Roche Diagnostics), and denatured at 95°C for 3 min prior to transfer to the slide surface. Hybridizations were conducted for 16 h at 42°C in a hybridization chamber (Scienion AG). After washing (once with 0.2×SSC-0.1% [wt/vol] sodium dodecyl sulfate and twice with 0.2×SSC for 5 min) and drying by centrifugation, the slides were scanned with a GMS 418 microarray scanner (MWG Biotech AG). Image processing and grid placement were achieved with the AIDA image analysis software package (Raytest).

### Microarray Data Analysis

The raw fluorescence intensity data were processed and normalised using quantile normalisation (log_2_; [56]). Only probes that could be associated with a unique EST were used for the bioinformatic analyses. Differential expression between experimental groups was assessed by using linear models and empirical Bayes unpaired moderated *t*-statistics. The expression ratios and their corresponding moderated bayes *p*-values, were obtained for the studied pairwise comparisons (air *vs*. water incubated embryos at different time points). Determinations of false discovery rates (FDR) were performed using the Benjamini-Hochberg procedure [57]. Heatmaps of expression profiles were made showing hierarchical clustering using euclidean distances. All computations were done using Limma R-package software [58]. The significance level was set at *p*<0.01. The microarray data have been deposited in NCBI’s Gene Expression Omnibus (GEO) [59] and are accessible through GEO Series accession number GSE19424 (http://www.ncbi.nlm.nih.gov/geo/query/acc.cgi?acc=GSE19424).

### Validation of Microarray Results by Real-time Reverse Transcription PCR

A subset of differentially expressed genes (*n* = 20) were selected to validate the fold changes values detected by microarray using quantitative real-time reverse transcription PCR (qPCR) on the same RNA samples than those employed for the microarray. An aliquot corresponding to 0.5 µg was reverse transcribed using 0.5 µg oligo(dT)_17_, 1 mM dNTPs, 40 IU RNase inhibitor, and 10 IU MMLuV-RT enzyme (Roche), for 1.5 h at 42°C. qPCR was performed with an ABI PRISM 7900HT Sequence Detection System (Applied Biosystems) using Power SYBRGreen PCR Master Mix (Applied Biosystems). Amplification was in a final volume of 20 µl, containing 10 µl of the master mix, 2 µl of cDNA diluted 1∶10 and 0.2 µM of each gene specific oligonucleotide primer. Primer3 software [60] was used to design primers. *F. heteroclitus* ornithine decarboxylase (*odc*) was used as a reference gene, since its expression between experimental samples did not show significant differences (data not shown; [27]). The cycle was set as follows: activation for 120 s at 50°C and initial denaturation for 10 min at 95°C, followed by 40 cycles at 95°C for 10 s (denaturation) and 63°C for 60 s (annealing and extension). A final step, decreasing the temperature from 95°C to 60°C and increasing from 60°C to 95°C, was used to determine the melting curve. Negative control samples were also run in which the template was not added. The threshold cycle number (Ct) was determined for all PCR reactions. Formation of primer dimers was checked by adding water instead of cDNA in the reaction mixture. The linear correlation between the quantitative-PCR and microarray data was performed based on the log mean values. Log_2_ fold changes were assessed using -(T-C) where T = delta Ct of gene of air incubated embryos and C = delta Ct of gene of water immersed embryos. The Spearman’s rank correlation with microarray was determined using SigmaPlot version 12.0 (SPSS Inc.).

## Results and Discussion

### Characterization of a Multi-stage *F. heteroclitus* Embryo Transcriptome

To obtain a representative picture of the adaptive transcriptome response of embryonic *F. heteroclitus*, a total of 16,896 clones were Sanger-sequenced from a normalized embryo cDNA library constructed from embryos at different stages incubated in water or in air. After extensive quality control, 15,686 ESTs were identified, which represent 92.8% of the total ESTs sequenced. Clustering analysis yielded 2,251 contigs (containing 40.5% of the input sequences) and 9,337 singletons (representing 59.5% of the input sequences), resulting in the identification of 11,588 different transcripts (corresponding to 73.9% of the whole sequencing program) (Table 1). Most of the contigs presented two members, although almost one third of the total number of contigs (37.9%) included more than two members (Table 1 and Figure S1A). The average number of ESTs per contig was 284, and the average length of the assembled sequences was 800–900 bp (Figure S1B). Almost 40% of the assembled sequences were 500–700 bp in length and the remaining ones were longer. The longest contig was 2204 bp in length. Most of the 9,337 singletons were 500–700 bp in length.

**Table 1:** Features of the *Fundulus heteroclitus* multi-stage embryo EST databank constructed in this study.

	No.	%
Total ESTs	15,686	–
Unigenes	11,588	73.9
Contigs	2,251	40.5
Contigs with >2 members	853	37.9
Singletons	9,337	59.5
Redundancy	6,349	40.5
BLAST hits	4,061	35.0

The EST annotation and GO analyses provided product or gene names for 4,061 sequences (35% of the total sequences), whereas 7,527 ESTs (65%) did not show significant similarity to any known protein in public databases. This low number of annotated ESTs is possibly partially caused by the relatively low number of deposited sequences from *F. heteroclitus* in GenBank (∼90,000 sequences) when compared to other model species such as the zebrafish (*Danio rerio*; ∼2,000,000 sequences; http://www.ncbi.nlm.nih.gov/sites). Other causes may be the presence of remaining genomic contamination during RNA extraction and long 5′ or 3′ UTRs of some transcripts. In any case, the most abundant ESTs (represented 10 times or more) in our normalized library of killifish embryos corresponded to cytoskeletal proteins (myosin, actin-related protein, thymosin), metabolic enzymes (fructose-bisphosphate aldolase, glutamate dehydrogenase, ATP synthesis-related proteins), protein biosynthesis (eukaryotic translation initiation factor 4A1), and S100 calcium binding protein V2 (Table S1). Interestingly, genes potentially involved in responses to hypoxia (cytochrome c oxidase subunit 4 isoform 1), oxidative stress (glutathione peroxidase 4) and ion transport (FXYD domain containing ion transport regulator 11b, voltage-dependent anion channel 3) were also highly represented (Table S1).

The majority of the functionally annotated sequences had GO assignments for molecular function (737 ESTs), cellular component (808 ESTs) and biological process (621 ESTs) categories (Figure S1C). Sequences with GO terms corresponding to molecular function fell into 10 categories, the annotation being well distributed with more sequences related to catalytic activity (31%), binding function (26%), structural molecular activity (13%), signal transduction (11%) or transporter activity (10%). Other functions were less represented (>5%) such as motor activity, transcription regulation, antioxidant function or translation regulator activity. Regarding biological process, most of the sequences (91%) were related to metabolic processes, including anabolism and catabolism as well as DNA repair and replication, and protein synthesis and degradation. The category of the cellular component most represented was the extracellular region (68%), i.e. the space external to the outermost structure of a cell, followed by protein complex (19%) and synapse (9%). The results of the GO analysis thus suggest that most of the genes being enriched during *F. heteroclitus* embryogenesis [40] were well represented in our library.

### Time Course of Transcriptome Changes in Response to Aerial Incubation

**Figure 1:**
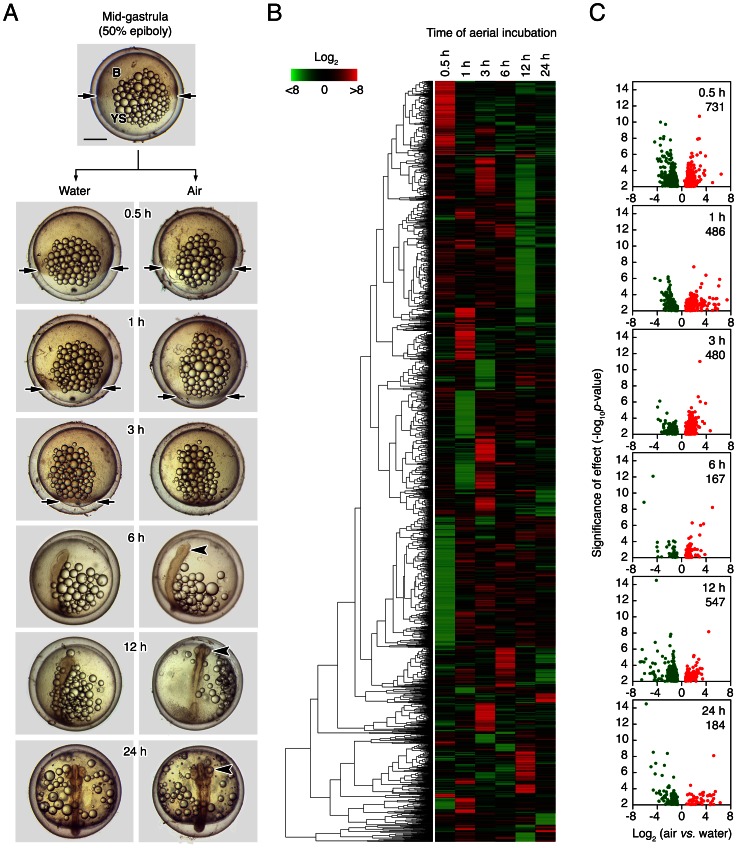
Stages and differential expression of *Fundulus heteroclitus* embryos incubated in water or exposed to air. (**A**) Representative photomicrographs of mid-gastrula embryos (50% epiboly), and of developing embryos after 0.5, 1, 3, 6, 12 and 24 h of aerial incubation at ∼100% RH as indicated. Note that aerial incubation enhances the rate of development, as indicated by the acceleration of epiboly (arrows) and the differentiation of eye and otic vesicles (arrowheads) in air exposed embryos with respect water submerged embryos from the same batch. (**B**) Hierarchical clustering of 2,381 putative genes that changed significantly (*p*<0.01) between air and water incubated embryos at each time point shown in A. Distances are measured using euclidean distance. Results are expressed as a matrix view of expression data where rows represent sequences and columns represent time points. The intensity of each colour denotes the standardized ratio between each value and the average expression of each sequence across all time points. Red pixels correspond to an increased abundance of mRNA, whereas green pixels indicate decreased mRNA levels. The list of all significantly regulated genes is given in Table S2. (**C**) Pairwise differences at each time point during aerial incubation. Significances of differences as −log_10_(*p*-values) are plotted against log_2_ differences in expression (air *vs*. water). Colors in these plots correspond to the colors of the gene tree in panel 1B.

In the present study, we aimed at investigating changes in the transcriptome of *F. heteroclitus* embryos exposed to air *vs*. embryos continuously submerged in seawater. Previously, we have shown that aerial incubation of killifish embryos at the blastula stage under 100% RH enhances the developmental rate with respect water-immersed embryos, six days of air exposure being able to advance the mean hatching time by four days [27]. Under these conditions, it seems that embryos can transduce the desiccating environment into molecular responses, such as the down-regulation of *aqp3a* both at the RNA and protein levels [27]. In a recent study, Chuaypanang et al. [26] showed that mid-stage *F. heteroclitus* embryos exhibit the highest degree of desiccation resistance, and therefore we first tested whether aerial incubation of mid-gastrula embryos can advance development. Figure 1A shows that 1 h of aerial incubation can indeed enhance the rate of epiboly in developing mid-gastrula embryos, since in these embryos a slight advancement of the epiboly process is clearly observed (Figure 1A, arrows). By approximately 3 h, the gastrulation process is completed in aerially incubated embryos, whereas this process is delayed in water-immersed embryos. At longer times of aerial exposure (6 to 24 h), the differences in the developmental rates between air and water incubated embryos are more evident at the level of the differentiation of the eye and otic vesicles (Figure 1A, arrowheads), as well as in the initiation of skin pigmentation. These differences are also reflected in the pattern of oil droplets within the yolk sac, which are smaller and appear more dispersed in aerially incubated embryos (Figure 1A), likely suggesting an enhanced lipid mobilization process.

The above observations indicate that mid-gastrula embryos are able to accelerate development in response to aerial incubation, and these embryos may also show high desiccation resistance [26]. To investigate whether the transcriptome of these embryos is altered due to aerial incubation, we determined changes in gene expression after 30 min and 1, 3, 6, 12 and 24 h of aerial exposure by cDNA microarray analysis. The microarray used was constructed from the EST collection obtained from the multi-stage *F. heteroclitus* embryo cDNA library, and contained 11,588 unigenes from which 4,061 sequences could be annotated. Although this number of genes is not the full complement of genes expressed in vertebrates, it may provide a statistically robust measure of differences between air and water incubated *F. heteroclitus* embryos, and therefore may still be useful for exploring embryo responses to the altered environment [40].

**Figure 2:**
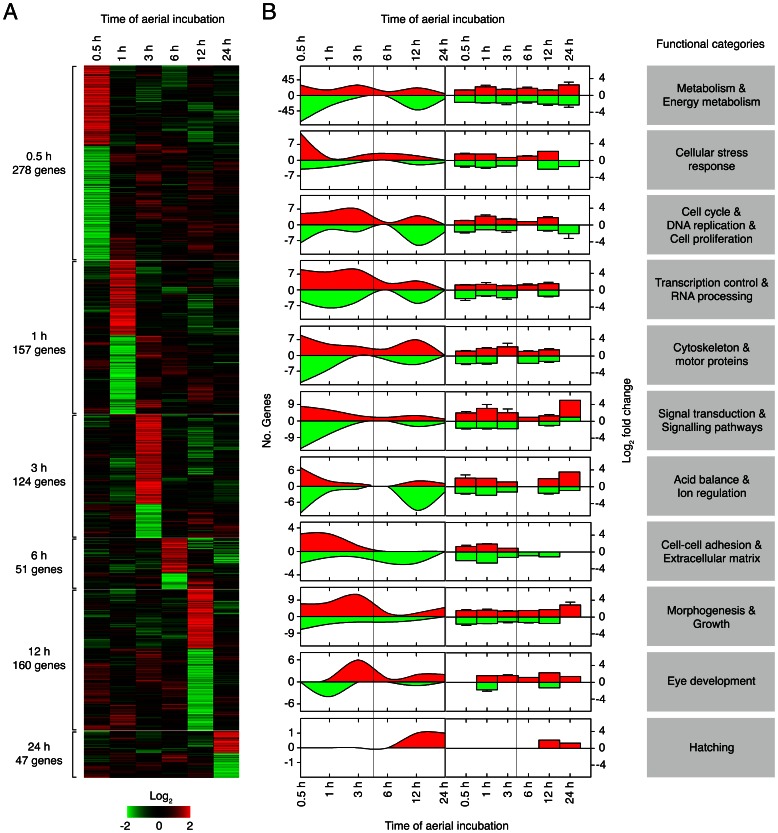
GO enrichment results of transcripts in aerially incubated *Fundulus heteroclitus* embryos. (**A**) Heatmap of annotated genes differentially expressed at different times during aerial incubation (0.5, 1, 3, 6, 12 and 24 h) ordered by time of significant (*p*<0.01) regulation. (**B**) Left panels show the number of significantly up- and down-regulated annotated genes (red and green, respectively) after 0.5, 1, 3, 6, 12 and 24 h of aerial incubation. Right panels show the log_2_ fold changes (mean, ± SEM when possible) at each time point of genes grouped according to functional categories as indicated on the right.

The expression of a total of 2,381 different genes (21% of 11,588), from which 806 genes could be annotated, was found to differ significantly (*p*<0.01) between water and air incubated embryos during the time course of aerial incubation, in which embryos develop from stage 17 up to approximately stage 21 [42] (Figure 1A and B; Table S2). qPCR validation verified expression differences on a subset of differentially expressed genes (Figure S2). Throughout the same stages during *F. heteroclitus* embryogenesis, Bozinovic et al. [40] reported differential expression of 217 (annotated) genes, thus a lower number of genes than in the present study. Also, in this previous study log_2_ fold changes in expression for most genes were found around ±3 or lower, whereas in our experiment these values tend to be higher (Figure 1C). These observations may indicate that the altered transcriptome associated to the progression of development under aerial incubation may be a minor event when compared to the specific response to the desiccation challenge. Interestingly, the highest number of differentially expressed genes (731, 31% of 2,381) was noted after only 30 min of air exposure, with most of the genes (61%) being down-regulated and showing slightly higher significant differences (Figure 1C). After 1 h, about the same number of genes are up- and down-regulated with similar significances (20% in total of 2,381), but the expression ratios tend to be higher for the up-regulated genes, suggesting that activation of embryonic gene expression may become more dominant at this time in the aerially incubated embryos (Figure 1C). By 3 and 6 h of aerial incubation, most of the 480 (20% of 2,381) and 167 (7% of 2,381) differentially expressed genes, respectively, are up-regulated (72% and 68%, respectively) with slightly higher significances, whereas at 12 and 24 h another large wave of 547 regulated genes (23% of 2,381), in which most of them (74%) are down-regulated, is observed (Figure 1C). Finally, at 24 h the number of differentially expressed genes is reduced again (184; 8% of 2,381) and most genes (72%) and significant differences show decreases in expression (Figure 1C). When considering only the annotated genes, the analysis also shows that the largest wave of differentially expressed genes occurs within the first 3 h of air exposure (559 genes, 68% of total genes), half of these genes (278 genes, 34% of total genes) being regulated already at 30 min (Figure 2A). Therefore, these findings support our hypothesis that by inducing rapid transcriptomic responses *F. heteroclitus* embryos are able to adapt to environmental desiccation conditions, even to those that may not impose an extreme dehydration stress.

The putative function of the 806 differentially expressed genes annotated during aerial incubation was further investigated by GO analysis to obtain a first assessment of potential physiological processes involved (Figure 2). Most of the annotated ESTs (83%) had GO assignments, and many of those had 3–6 assignments each (91%). When genes were grouped according to their putative roles in major biological processes, those involved in metabolic processes represent the largest population (44%), these genes being more regulated at 30 min, 3 h and 12 h during aerial incubation (Figure 2B). Most of the metabolic genes (59%) were down-regulated, although they showed similar differences in expression than those up-regulated. Interestingly, genes involved in cellular stress, signaling pathways, cytoskeleton and motor proteins, and acid and ion regulation, are more abundant specifically within the first 30 min of aerial incubation, with those related to stress being up-regulated in most cases (Figure 2B). Other genes involved in cell cycle and related functions (DNA replication and cell proliferation), transcriptional control and RNA processing, and cell-cell adhesion and extracellular matrix, start also to be regulated at 30 min but the number of these genes appears to be higher at later times of air exposure (Figure 2B). The transcription-related genes, however, remain up-regulated during the 3 h of air exposure, suggesting that embryonic gene expression may be predominantly enhanced around this time. Accordingly, up-regulated genes potentially involved in morphogenesis and growth tend to be more abundant by 3 h, with maximum expression ratios at 24 h of aerial exposure (Figure 2B). Among these genes, those likely engaged in eye development start to be down-regulated at 1 h of air exposure, whereas they are predominantly up-regulated from 3 h onwards, which coincides with the first detection of eye and otic vesicle differentiation at 6 h in aerially incubated embryos (Figure 1A). In contrast, the *F. heteroclitus* gene encoding the low choriolytic enzyme (*flce*), which together with the high choriolytic enzyme (*fhce*) is likely involved in the digestion of chorion during hatching [61], is up-regulated at 12 and 24 h (Figure 2B). Such early activation of the *flce* gene in air-incubated embryos with respect those maintained in water is thus consistent with the advanced hatching of these embryos when cued by water immersion [27], which possibly also suggest and earlier formation of the hatching gland.

### Desiccation Sensing and Analysis of the Early Transcriptomic Response

The analysis of putative functions of the differentially expressed genes suggest that within the 3 h period of aerial incubation, *F. heteroclitus* embryos might show two phases in the regulatory response to desiccation conditions, in a similar fashion to that described in the adult gills in response to an extremely short (i.e. 6 h) osmotic shock [38]. According to this model, the first phase in embryos may involve a rapid (within ∼30 min) sensing of adverse external conditions, which triggers the activation of specific signaling pathways and mechanisms to cope with the stress (possibly desiccation, thermal as well as osmotic), and alterations in transcription and cell organization. These events may trigger a second phase (from ∼3 h onwards), which is primarily associated with the regulation of many different effectors, functioning to enhance embryonic development. These two phases however seem to overlap to some extent.

We selected five groups of genes involved in (1) signaling pathways, (2) stress response, (3) protein metabolism, (4) lipid and yolk metabolism, and (5) morphogenesis and growth as examples, and discuss the expression patterns of these genes in relation to their potential biological significance in the sensing and immediate response of embryos to the desiccation cue.

#### Signaling pathways

**Figure 3:**
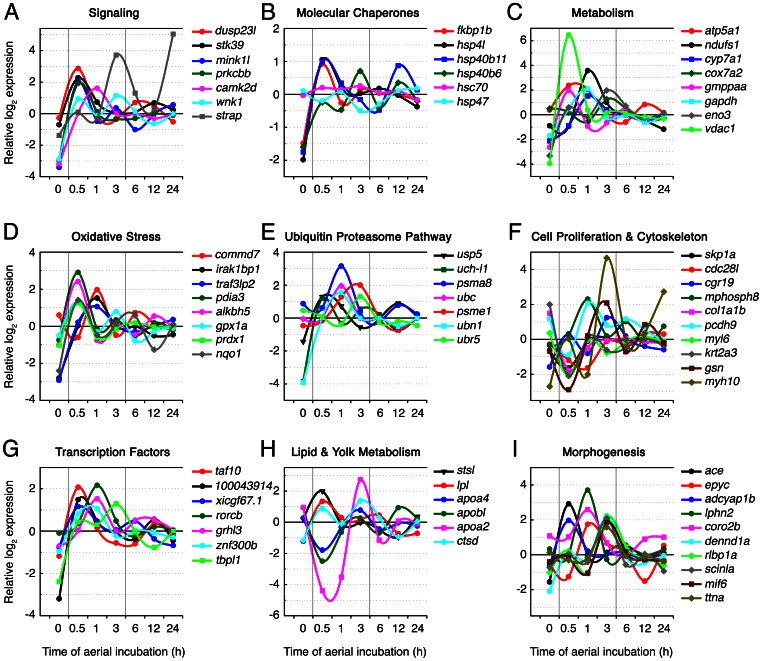
Time-course of log_2_ gene expression change on aerial incubation challenge in *Fundulus heteroclitus* embryos, partitioned by putative functional category. Representative genes of each category are shown. Log_2_ values at time 0 correspond to differences in expression in mid-gastrula embryos *vs*. 2–4 cells stage embryos. (**A**) *wnk1*, serine/threonine-protein kinase WNK1-like; *strap*, serine/threonine kinase receptor associated protein; *dusp23l*, novel protein similar to vertebrate dual specificity phosphatase 23; *stk39*, serine/threonine kinase 39, STE20/SPS1 yeast homolog; *mink1l*, novel protein similar to vertebrate misshapen-like kinase 1; *prkcbb*, protein kinase C beta 1; *camk2d*, calcium/calmodulin-dependent protein kinase type II delta chain. (**B**) *fkbp1b*, peptidyl-prolyl cis-trans isomerase; *hsp4l*, heat shock protein 4, like; *hsp40b11*, heat schok protein 40 B member 11; *hsp40b6*, DnaJ-like subfamily B member 6; *hsc70*, heat shock cognate 70; *hsp47*, heat shock protein 47. (**C**) *atp5a1*, ATP synthase subunit alpha; *ndufs1*, NADH dehydrogenase (ubiquinone) Fe-S protein 1, 75 kDa (NADH-coenzyme Q reductase); *cyp7a1*, cholesterol 7 alpha-hydroxylase; *cox7a2*, cytochrome c oxidase, subunit VIIa 2; *gmppaa*, mannose-1-phosphate guanyltransferase alpha-A; *gapdh*, glyceraldehyde 3-phosphate dehydrogenase; *eno3*, beta enolase; *vdac1*, voltage-dependent anion channel 1. (**D**) *commd7*, COMM domain-containing protein 7; *irak1bp1*, interleukin-1 receptor-associated kinase 1-binding protein 1; *traf3lp2*, TRAF3 interacting protein 2; *pdia3*, disulfide-isomerase A3; *alkbh5*, alkylation repair homolog 5; *gpx1a*, glutathione peroxidase; *prdx1*, peroxiredoxin 1; *nqo1*, NAD(P)H dehydrogenase quinone 1. (**E**) *usp5*, ubiquitin specific protease 5; uch-l1, ubiquitin C-terminal hydrolase; *psma8*, proteasome subunit alpha type; *ubc*, ubiquitin carrier protein; *psme1*, proteasome activator complex subunit 1; *ubn1*, ubinuclein 1; *ubr5*, ubiquitin protein ligase E3 component n-recognin 5. (**F**) *skp1a*, S-phase kinase-associated protein 1A; *cdc28l*, novel protein similar to vertebrate CDC28 protein kinase family; *cgr19*, novel protein similar to rodent cell growth regulator with RING finger domain 1; *mphosph8*, M-phase phosphoprotein 8; *col1a1b*, collagen type I alpha 3 chain; *pcdh9*, protocadherin-9; *myl6*, Myosin light polypeptide 6; *krt2a3*, Type II keratin E3; *gsn*, Gelsolin; *myh10*, Myosin 10. (**G**) *taf10*, transcription initiation factor TFIID subunit 10; *100043914*, novel KRAB box and zinc finger, C2H2 type domain containing protein; *xicgf67.1*, gastrula zinc finger protein XlCGF67.1; *rorcb*, RORgamma-B; *grhl3*, Grainyhead-like protein 3 homolog; *znf300b*, zinc finger protein 300-B; *tbpl1*, TATA box-binding protein-like protein 1. (**H**) *stsl*, novel protein similar to human steroid sulfatase (microsomal); *lpl*, lipoprotein lipase; *apoa4*, apolipoprotein A-IV; *apobl*, novel protein similar to vertebrate apolipoprotein B; *apoa2*, apolipoprotein A2; *ctsd*, cathepsin D. (**I**) *ace*, Angiotensin I converting enzyme; *epyc*, Epiphycan; *adcyap1b*, growth hormone releasing hormone/pituitary adenylate cyclase- activating; *lphn2*, Latrophilin 2; *coro2b*, Coronin-2; *dennd1a*, DENN domain-containing protein 1A; *rlbp1a*, cellular retinaldehyde-binding protein a; *scinla*, Gelsolin; *mif6*, myogenic factor 6; *ttna*, novel protein similar to human titin.

One well-known group of proteins involved in the transduction of sensor signals to stress response genes are the mitogen-activated protein kinases (MAPKs), which upon activation by phosphorylation, either enter the nucleus and regulate gene expression or remain in the cytoplasm and phosphorylate substrates [62]. All three types of MAPKs (ERK, p38 and JNK) play a role during desiccation, oxidative and osmotic stress in a wide range of organisms, regardless of whether they are able to reach an anhydrobiotic stage and survive severe desiccation conditions [63], [64], [65]. In *F. heteroclitus* mid-gastrula embryos, MAPKs may also be involved in the sensing and transduction of desiccation since we observed that expression of genes that can potentially participate in the p38 MAPK and SAPK/JNK (*dusp23-like*, *stk39*, *mink1-like*), and MAPK/ERK (*prkcbb*, *camk2d*), signaling pathways is strongly and transiently enhanced after 30 min-1 h of aerial incubation (Figure 3A). In addition, genes related to different NF-κB signaling pathways, which are known to be involved in the cell protection to apoptosis, DNA damage and oxidative stress [66], were also elevated at 1 h (see below). The importance of phosphorylation cascades as major signal transduction pathways possibly involved in the compensatory response of killifish embryos to desiccation is also illustrated by other genes, such as the Serine/threonine-protein kinase WNK1-like (*wnk1*) and the Serine/threonine kinase receptor associated protein (*strap*). The expression of these latter genes is strongly up-regulated at 3 and 24 h or aerial incubation, respectively (Figure 3A). Altogether, these data suggest that killifish embryos are able to sense and respond appropriately to desiccation conditions, even to those that do not impose water loss and are apparently readily tolerated [26], [27].

#### Molecular chaperones

Many proteins of the cellular chaperone systems are expressed ubiquitously in the normal cell state to aid in the folding of native polypeptides and their translocation to different cellular compartments [67]. Synthesis of stress-induced molecular chaperones, such as heat shock proteins (Hsps), is known to be up-regulated during the stress response to prevent misfolding or aggregation of proteins and to regulate their degradation [68], [69]. The fact that elements of the molecular chaperone system are up-regulated in aerially incubated killifish embryos (Figure 3B) is consistent with the supposition that these elements may help to stabilize cellular proteins under desiccation and osmotic stress [26]. This is exemplified by the up-regulation within 3 h of air exposure of *hsp40* and the co-chaperone peptidyl-prolyl cis-trans isomerase (*hsp40b6*), which are induced by heat shock [70], [71], although with relatively low log_2_ expression ratios <1.5. In addition, the expression of the *F. heteroclitus* ortholog of the human protein highly similar to heat shock 70 kDa protein 4 (*hsp4-like*), also found in zebrafish and carp (http://www.uniprot.org/) but of unknown function, also increases by ∼1-fold at 30 min. However, two other molecular chaperones encoding genes *hsc70* and *hsp47*, which were represented in the microarray, showed no significant changes after desiccation stress (Figure 3B). In *F. heteroclitus*, the Hsc70 is a constitutively expressed isoform of Hsp70 that may be induced in adult gills by heat shock in Southern populations but not in Northern populations of North America [72]. Therefore, in terms of *hsc70* expression, the absence of response to air incubation in our experiments may be related to adaptive differences in gene expression to thermal conditions of different killifish populations [30]. However, Chuaypanang [73] found that *hsc70*, *hsp70*, *hsp90α* and *hsp90β* expression in *F. heteroclitus* embryos is largely constitutive with no major changes after desiccation stress compared with controls, and no significant differences among embryo ages. These findings suggest that Hsp40-related proteins, rather than Hsp70, and other small Hsps might be involved during desiccation resistance in killifish embryos. Indeed, small Hsps may play a particularly important role in dormant organisms, as they act as ATP-independent chaperones, thus preventing protein aggregation in stressful conditions without the expenditure of important ATP reserves [5], [74]. This would be consistent with the large amounts of ATP that may be required in killifish embryos to activate morphogenesis and growth shortly after sensing and responding to the desiccation cue (see below).

#### Oxidative stress

In killifish embryos, one benefit of aerial exposure during delayed hatching is thought to be the increased oxygen availability, which enhances the rate of development and may result in larger hatchlings [27], [28]. It has been suggested that the respiratory demands of developing killifish embryos are fulfilled by the oxygen supply from the air, which can be at least 20-fold higher than in water, whereas hatching is cued when the metabolic rate of the embryo exceeds a limit set by the diffusion of oxygen through the water column [18]. The enhanced developmental rates of embryos exposed to air is however likely associated with an increased metabolic rate to sustain signal transduction pathways, the cell defense to desiccation and heat stress, and the activation of embryonic development. These processes, together with the hyperoxia caused by the direct exposure of embryos to air, may in turn generate mitochondrial reactive oxygen species (ROS) that are toxic to the cell [66], [75]. This scenario of oxidative stress is most likely to occur in aerially incubated mid-gastrula killifish embryos since a number of metabolic genes are rapidly up-regulated (Figure 3C; Table S2), such as ATPases (*atp5a1*), glycolityic (*gapdh*, *eno3*) and sugar biosynthesis (*gmppaa*) enzymes. High expression of mitochondrial electron transport chain complexes (*ndufs1*, *cyp7a1*, *cox7a2*) is also observed which are the greatest source of ROS since the reactions that occur during oxidative phosphorylation processes frequently lose electrons that react with molecular oxygen to produce ROS [66]. Elevated metabolic activity of aerially incubated embryos may also be supported by the strong up-regulation (by >6-fold; Figure 3C) of transcripts encoding the voltage dependent anion channel 1 (porin), a protein of the outer mitochondrial membrane that forms a pore for the entry and exit of metabolites, including ATP, between this organelle and the cytosol [76]. Accordingly, we observed a rapid activation of several genes that are well known to be involved in the cell response to oxidative stress such as peroxiredoxins, glutathione peroxidases, NAD(P)H dehydrogenase quinone 1 (*nqo1*), disulfide-isomerase A3 (*pdia3*), ferritin lower subunit (*ftl*) and manganese superoxide dismutase (*mnsod*) [66], [77] (Figure 3D; Table S2). Intriguingly, we also found that the alkylation repair homolog 5 ortholog (*alkbh5*), which is a 2-oxoglutarate-dependent oxygenase that may modulate patterns of histone methylation in hypoxic cells [78], is highly up-regulated (2.42-fold increase) within 30 min of aerial incubation.

As mentioned earlier, some genes that can potentially participate or regulate both canonical and non-canonical NF-κB signaling pathways (*commd7*, *irak1bp1*, *traf3lp2*) are activated in aerially incubated embryos at 1 h (Figure 3D). These pathways activate the NF-κB transcription factor, which increases the expression of several antioxidant proteins [66]. Interestingly, some of the typical antioxidant target genes of NF-κB are up-regulated in embryos within the first 3 h of aerial incubation, such as the Mnsod mitochondrial enzyme that protects cells from oxidative stress by converting^.^O2^−^ into H_2_O_2_ [79], the cytoplasmic Gpx1 enzyme that catalyzes the conversion of H_2_O_2_ into water using glutathione as a substrate [80], or the FAD-binding Nqo1 reductase that prevents the one electron reduction of quinones that produces radical species [81]. These observations could suggest that activation of NF-κB signaling pathways contributes to the response of killifish embryos to oxidative stress.

#### Protein metabolism

During the first 3 h of aerial exposure, a high number of genes related to protein metabolism, particularly with catabolic processes, transport and synthesis appear to be up-regulated. Among these genes, we found higher expression of several proteases such as calpain, Adamts, or the aminopeptidase Npepps, which is preceded by a considerable down-regulation (∼2.4-fold) of some proteinase inhibitors as serpin and cystatins (Table S2). However, genes related to the ubiquitin proteasome degradation pathway, devoted to targeted degradation of most proteins including cue regulators of cell cycle, transcription regulators or misfolded proteins [82], [83], were the most represented, including the ubiquitin specific protease 5 (*usp5*) and ubiquitin C-terminal hydrolase L1 (*uch-l1*) (Figure 3E). By 30 min, we also observed a transient down-regulation of genes related to cell cycle and cell proliferation, the cytoskeleton, and cell-cell adhesion and extracellular matrix, and the up-regulation of many transcription factors (Figure 3F and G; Table S2). Genes related to these functional categories are activated later at 1 and 3 h of aerial exposure or remain up-regulated. Taken together, these data may point to an initial phase of tissue remodeling in aerially incubated embryos, where ubiquitin-mediated proteolysis might play a role [84], which overlaps, at least in part, with the activation of embryonic differentiation. In zebrafish embryos, the ubiquitin proteasome proteolytic pathway has been involved in the BMP pathway which plays a main role in determining the ventral cell fates [85], the *notch* signaling pathway [86], as well as in the degradation of paired-like homeobox gene *vsx1* [87]. Therefore, the early transcriptomic response of aerially incubated killifish embryos may resemble the cell stabilization and remodeling phase described in adult gills after hyposmotic shock, during which many genes associated with cell cycle regulation, cell growth, cell morphology, and the cytoskeleton, are transiently or permanently up-regulated [38].

#### Lipid metabolism and yolk processing

The expression of genes coding for proteins related to lipid and lipoprotein metabolism are among the most regulated in embryos during the first 3 h of aerial exposure (Figure 3H). In fish embryos, lipid and yolk (i.e. vitellogenin derived yolk proteins) reserves are stored within the yolk sac and represent the main energy source for embryo nourishment until external feeding. The yolk syncytial layer (YSL) or periblast consists of a vitellolysis zone and a cytoplasmic zone surrounding the yolk, and is therefore considered to play an integral role in the absorption of yolk in fish [88]. Accordingly, it is known that the YSL express a number of apolipoproteins, cysteine or aspartic proteases (cathepsins), and lipases, which allow yolk protein hydrolysis and lipid mobilization and absorption [89], [90], [91], [92], [93]. In our study, we found that the lipoprotein lipase encoding gene (*lpl*) is rapidly up-regulated at 30 min of aerial exposure which would be consistent with the immediate requirement of lipid mobilization to sustain enhanced embryonic development. Lipoprotein lipase is a triglyceride lipase widely expressed in a broad range of animals, including mammals and fish, and is involved in the hydrolysis of triglycerides of circulating chylomicrons and very low-density lipoproteins [94], [95], [96]. In killifish, the expression of *lpl* seems to occur much earlier than in other teleosts, since in rainbow trout (*Oncorhynchus mykiss*) no transcript is detected before stage E9 [97], and in zebrafish the expression starts in the periderm at the 14–19 somites stage [98]. Intriguingly, the elevated *lpl* expression in aerially incubated killifish embryos is concomitant with a transient down-regulation of several genes encoding apolipoproteins (*apoa2*, *apobl* and *apoa4*), as well as of fatty acid binding proteins and transporters (*fabp6*, *h-fabp*, *slc27a1*; Table S2), which are essential for lipid transport and mobilization during development [92], [99]. These observations are therefore surprising but they may suggest that lipid hydrolytic processes, rather than transport mechanisms, are the immediate reaction to aerial exposure, possibly to sustain an increased metabolic rate. By 3 h of aerial incubation, lipid transport and yolk protein processing seems to be activated since apolipoprotein as well as cathepsin D (*ctsd*) genes are up-regulated. Some studies in teleosts have implicated the aspartic protease Ctsd in the processing of yolk proteins [89], [97], [100], and a recent study in zebrafish has shown the critical role of Ctsd in the retinal pigment epithelium, swim-bladder formation and growth [101]. Therefore, the subsequent response of embryos seems to be the mobilization of yolk reserves and the increase of circulating apolipoproteins as a source of fatty acids, which together with the stimulation of protein turn-over (synthesis, post-translational modification, and transport; see above), are possibly required to enhance embryo development.

An interesting observation in our study is the relatively strong up-regulation (by ∼2-fold) of the steryl-sulfatase-like (*stsl*) enzyme (Figure 3H), which is involved in the conversion of sulfated steroid precursors to estrogens during pregnancy [102]. In a recent study, Pri-Tal et al. [103] reported that *A. limnaeus* embryos that escape diapause II show higher levels of androstenedione (estrogen precursor) and estradiol that embryos entering diapause, suggesting that that steroid hormonal signaling is involved in the regulation of developmental trajectories in this species. Sulfatation of the 3β-hydroxyl group of a steroid may transforms a liposoluble steroid into a water soluble molecule, and therefore modifies its subcellular localization and biological activities [104]. Although in our experiments we could not identify the activation of estrogen signaling pathways in aerial incubated killifish embryos, a potential high sulfatase activity in these embryos may suggest that steroid hormones might be involved in the pathway triggering the acceleration of development as in *A. limnaeus* escape embryos. Future studies will be necessary to test this hypothesis.

#### Morphogenesis and growth

The acceleration of development in aerial incubated embryos is illustrated by the up-regulation within the first 3 h of a number of genes potentially involved in different morphogenetic and growth processes (Figure 3I; Table S2). Most of these genes however are not activated until 3 h, which may reflect a second and rapid phase in the response of embryos to aerial exposure during which the major event seems to be the estimulation of development. Among the processes that appear to be activated are angiogenesis (*ace*), bone and cartilage formation (*epyc*), eye, brain and nervous system development (*adcyap1b*, *lphn2*, *coro2b*, *dennd1a*, *rlbp1a*, *scinla*), and muscle development (*mif6*, *ttna*).

### Conclusions

In summary, we used microarray analysis to identify alterations in the *F. heteroclitus* embryonic transcriptome in response to aerial incubation. The data revealed a very rapid and large transcriptomic response, including the differential regulation of at least 559 different genes within 3 h of air exposure. The observed changes in gene expression seem to define two overlapping phases during which the sensing of the environmental cue is followed by the activation of embryonic development. These findings therefore suggest that *F. heteroclitus* embryos possibly retain multiple transcriptional mechanisms, which allow them to rapidly acclimate to changing environmental conditions before hatching. Since the desiccation conditions employed in our study most likely do not impose severe water loss, and hence a dramatic dehydration stress, our results also imply that killifish embryos are able to sense and respond even to non-lethal environmental changes in water and/or oxygen availability. The present work however provides only a preliminary analysis of the changes of the killifish transcriptome during aerial incubation. Future studies using next-generation sequencing technologies or genome-wide microarray platforms, and also employing more detailed sampling (i.e. within minutes), will be very useful to obtain a deeper picture of the specific physiological mechanisms involved.

It seems noteworthy that aerial incubation of killifish embryos appears to differentially activate similar classes of transcripts that are reported to be expressed in previous studies on dormant forms of invertebrates and vertebrates, including desiccated models, in which metabolic activity is dramatically reduced [5]. Among these genes are those potentially involved in phosphorylation signaling pathways, oxidative stress or molecular chaperones [5], [65], [105], suggesting critical adaptations to survival in killifish embryos that may resemble to other species undergoing dormancy, diapause or desiccation. Thus, desiccation resistance during delayed hatching of killifish embryos may be an useful model to elucidate common and distinct pathways for the long term preservation of animal cells.

## Supporting Information

Figure S1 **Features of the multi-stage** ***F. heteroclitus*** **embryo cDNA library.** The normalized library was constructed from total RNA extracted from a pool of embryos at different developmental stages maintained in water or in air, and collected at different times after water addition. (**A**) Number of ESTs within each contig after assembly. (**B**) Mean contig EST length and mean length of singletons (in bp). (**C**) BLAST2GO categories of killifish ESTs.
(JPG)

Figure S2 **Log_2_ fold change correlations of selected genes detected by microarray and qPCR, and primer sequences.** (**A**) Log2 fold change detected by qPCR is depicted on the *x*-axis, and change detected by microarray is on the *y*-axis. The graph shows 120 comparisons of fold change in expression of 20 genes showing significant (*p*<0.01) differential expression at one or more time points during aerial incubation. Spearman’s rank correlation detected R = 0.88 (*p*<0.0001) correlation between microarray and qPCR detection of fold change. (**B**) Forward and reverse primer sequences for the 20 experimental and 1 reference genes used for qPCR to validate gene expression microarray results.
(TIF)

Table S1 **Largest EST clusters in the normalized** ***Fundulus heteroclitus*** **multi-stage embryo cDNA library.** The table lists the largest EST clusters (with >10 ESTs) in the normalized library and their annotation.
(PDF)

Table S2 **Genes changing significantly at different times in aerially incubated** ***Fundulus heteroclitus*** **embryos from the mid-gastrula stage with respect to embryos maintained submerged in water.** Data show log_2_ fold-differences in expression at different times (0.5, 1, 3, 6, 12 and 24 h) during aerial incubation and p-values. For Contigs, fold-changes and *p*-values are given for a representative probe.
(XLS)
