# Supplementary figures and images for: A Rapid Transcriptome Response Is Associated with Desiccation Resistance in Aerially-Exposed Killifish Embryos
Source: PLoS One. 2013 May 31;8(5):e64410. doi: 10.1371/journal.pone.0064410 (PMC3669298; doi:10.1371/journal.pone.0064410)

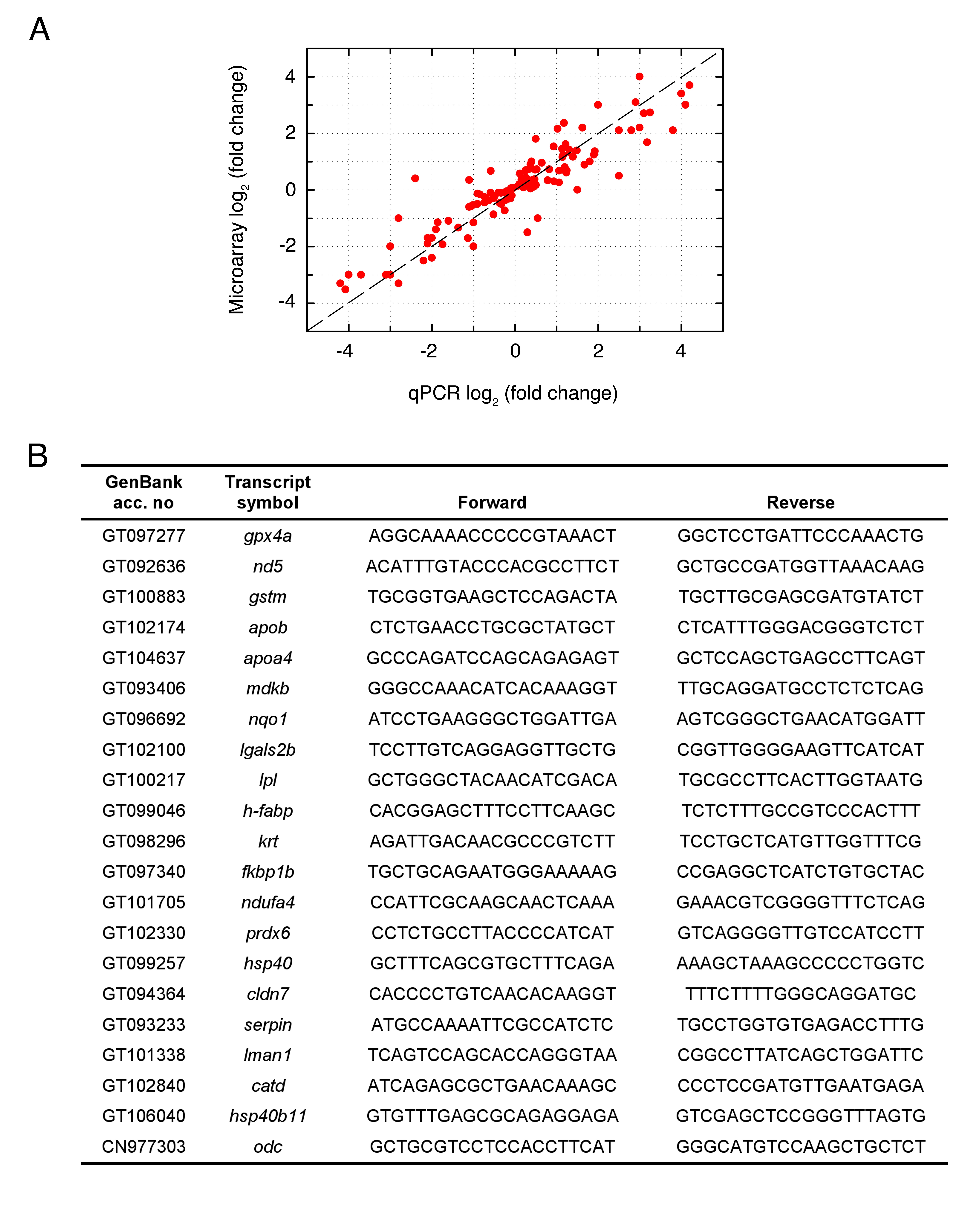

Supplement: Figure S2 — Log2 fold change correlations of selected genes detected by microarray and qPCR, and primer sequences. (A) Log2 fold change detected by qPCR is depicted on the x-axis, and change detected by microarray is on the y-axis. The graph shows 120 comparisons of fold change in expression of 20 genes showing significant (p<0.01) differential expression at one or more time points during aerial incubation. Spearman’s rank correlation detected R = 0.88 (p<0.0001) correlation between microarray and qPCR detection of fold change. (B) Forward and reverse primer sequences for the 20 experimental and 1 reference genes used for qPCR to validate gene expression microarray results. (TIF) [file pone.0064410.s002.tif]
