# Supplementary material for: A Rapid Transcriptome Response Is Associated with Desiccation Resistance in Aerially-Exposed Killifish Embryos
Source: PLoS One. 2013 May 31;8(5):e64410. doi: 10.1371/journal.pone.0064410 (PMC3669298; doi:10.1371/journal.pone.0064410)
Supplement: Table S1 — Largest EST clusters in the normalized Fundulus heteroclitus multi-stage embryo cDNA library. The table lists the largest EST clusters (with >10 ESTs) in the normalized library and their annotation. (PDF) [file pone.0064410.s003.pdf]

Table S1. Largest EST clusters (with ≥10 ESTs) in the normalized *Fundulus heteroclitus* embryo cDNA library

**Contig ID**	**GenBank hit acc. no** [a]	**Gene identification (species) of top BLAST hit** [a]	**BLAST E-value** [a]	**Length (% identity)** [b]	**No. ESTs**
sb010-CL7Contig1	Q6PHJ8	Myosin, light polypeptide 9, like (*Danio rerio*)	4e-85	153/159 (96%)	24
sb010-CL2Contig1	Q8WNT7	Fructose-1,6-bisphosphate aldolase A (*Macaca fascicularis*)	3e-103	203/204 (99%)	22
sb010-CL11Contig1	Q6DRD1	ATP synthase, H+ transporting, mitochondrial F1 complex, O subunit (*Danio rerio*)	2e-85	165/194 (85%)	13
sb010-CL13Contig1	C3KHA2	Cytochrome c oxidase subunit 4 isoform 1, mitochondrial precursor (*Anoplopoma fimbria*)	3e-30	123/153 (80%)	13
sb010-CL14Contig1	Q5XGH1	Eukaryotic translation initiation factor 4A1 (*Xenopus (Silurana) tropicalis*)	1e-152	290/307 (94%)	13
sb010-CL8Contig1	Q5SD51	NADH dehydrogenase subunit 5 (*Fundulus heteroclitus*)	0.0	490/522 (93%)	13
sb010-CL15Contig1		No significant similarity found			12
sb010-CL18Contig1	B5X5D9	Thymosin beta-a (*Salmo salar*)	6e-05	24/32 (75%)	12
sb010-CL20Contig1	B7U7V8	ATP synthase F0 subunit 6 (*Fundulus heteroclitus*)	2e-77	216/227 (95%)	12
sb010-CL6Contig1	Q6DHK6	Glutathione peroxidase 4 (*Danio rerio*)	2e-80	143/186 (76%)	12
sb010-CL26Contig1	B5DGJ9	S100 calcium binding protein V2-like (*Salmo salar*)	3e-29	62/100 (62%)	11
sb010-CL10Contig2	Q503Y1	Glutathione peroxidase 4b (*Danio rerio*)	1e-60	140/173 (80%)	10
sb010-CL16Contig1	Q6P0S2	Voltage-dependent anion channel 3 (*Danio rerio*)	1e-39	82/92 (89%)	10
sb010-CL21Contig1	B0S4R4	FXYD domain containing ion transport regulator 11b (*Salmo salar*)	3e-12	34/70 (48%)	10
sb010-CL22Contig1	Q8JHX9	Glutamate dehydrogenase 3 (*Oncorhynchus mykiss*)	6e-108	195/206 (94%)	10
sb010-CL25Contig1	C1BFF5	Actin-related protein 2/3 complex subunit 3 (*Oncorhynchus mykiss*)	2e-55	158/178 (88%)	10

[a]Most significant BLASTX hit is reported.
[b]Extent of BLASTX hit aligned region (in amino acids), and percent identity over the aligned region.
